# Relationship of high sensitivity C-reactive protein with presence and severity of coronary artery disease 

**DOI:** 10.12669/pjms.296.3302

**Published:** 2013

**Authors:** Syed Shahid Habib, Abeer A. Al Masri

**Affiliations:** 1 Dr. Syed Shahid Habib, Department of Physiology, College of Medicine & King Khalid University Hospital, King Saud University, Riyadh, Saudi Arabia.; 2 Dr. Abeer A. Al Masri, Department of Physiology, College of Medicine & King Khalid University Hospital, King Saud University, Riyadh, Saudi Arabia.

**Keywords:** High sensitivity C-reactive protein, Coronary artery disease, Gensiniscore, Vessel scores, Angiography

## Abstract

***Objective:*** Inflammation plays a key role in the pathogenesis of atherosclerosis. This study aimed to assess the relationship of serum inflammatory marker high sensitivity C Reactive protein (hsCRP), with the presence and severity of angiographically evaluated coronary artery disease (CAD).

***Methods:*** This study was conducted at departments of physiology and cardiology, College of Medicine & King Khalid University Hospital, King Saud University, Riyadh from August 2009 to March 2012. Eighty seven patients (57 males and 30 females) with angiographically evaluated CAD were studied. In all these patients CAD severity was assessed by Gensini scoring and vessel scoring. Control group consisted of 29 healthy subjects (17 males and 12 females). Fasting venous blood samples were analyzed for lipid profile and high sensitivity C-reactive protein (hsCRP).

***Results:*** There were non-significant differences in age, weight and BMI among healthy subjects and CAD patients. Comparison of lipid profile between control and CAD patients showed that CAD patients had significantly higher TG and significantly lower HDL levels compared to control subjects. CAD patients presented with significantly higherhsCRP levels than controls. Linear regression analysis between hsCRP and CAD severity determined by Gensini scores showed a significant positive correlation (r=0.423, p=0.018). Triple vessel disease patients had significantly higher hsCRP levels than one vessel and two vessel disease, while the difference was non significant between one and two vessel disease groups.

***Conclusions:*** These results suggest that patients with angiographically evaluated CAD have significantly higher levels of hsCRP levels compared to healthy individuals and are correlated with the presence & severity of CAD.

## INTRODUCTION

There is strong evidence that cardiovascular conditions are linked with inflammation. Likewise there is role of inflammation in the pathogenesis of atherosclerosis.^1^ This ultimately leads to the occurrence of acute cardiovascular events.^2^ The chronic inflammatory process in atherosclerosis usually results in an acute clinical event by plaque rupture and therefore causes acute coronary syndromes.^3^ Many large prospective trials have shown that the inflammatory biomarker high-sensitivity C-reactive protein (hsCRP) is an independent predictor of future cardiovascular events.^4^ Several studies from Europe and United States indicate that elevated levels of hsCRP among apparently healthy men and women are a strong predictor of future cardiovascular events.^5^^,^^6^ Addition of hsCRP to conventional risk factors acts as an independent significant predictor of cardiometabolic risk.^7^ hsCRP has been reported to be an independent significant predictor and a risk factor of cardiometabolic risk, with an additive value to metabolic syndrome components.^8^ It has a long-term predictive value in patients with diagnosed coronary artery disease (CAD) and angina pectoris.^9^^,^^10^ It is also useful as predictor in individuals with multiple risk factors.^11^ hsCRP not only is an important predictor of first myocardial infarction but also for recurrent coronary events.^12^^-^^14^ In most of the studies reported, the association of hsCRP with cardiovascular risk has been found to be highly significant in global risk-assessment programs.^15^ Little data is available regarding association of hsCRP with the presence and severity of CAD. To the best of our knowledge there are no studies correlating hsCRP levels in CAD with Gensini and vessel scoring of CAD severity.

This study aimed to assess the relationship of serum inflammatory marker high sensitivity C Reactive protein (hsCRP), with the presence and severity of angiographically evaluated coronary artery disease (CAD).

## METHODS

This project was conducted at Departments of Physiology and Cardiology, College of Medicine & King Khalid University Hospital, King Saud University, Riyadh from August 2009 to March 2012. The project was funded by the Deanship of Scientific Research at King Saud University through the cardiovascular research group project No. (RGP-VPP-016). The study protocol was approved by the ethical Committee of College of Medicine Research Center. In this cross sectional study 87 patients (57 males and 30 females) were studied who had undergone angiography and were found to have CAD. They were recruited from department of cardiology, King Khalid University Hospital, Riyadh. Control group consisted of 29 healthy subjects (17 males and 12 females) matched for age and BMI. They were in stable metabolic state and were not suffering from any acute or chronic inflammatory conditions that could affect hsCRP levels. They were free of any clinical manifestations of coronary, peripheral or cerebral artery disease by history, physical examination and electrocardiographic findings. Demographic data, family history and results of the coronary angiography were obtained from patient's files and filled in specially designed data collection form. Inclusion criteria consisted of adult patients of both sexes with ischemic heart disease who had attacks of angina or myocardial infarction and had undergone coronary angiography. Exclusion criteria included, acute or chronic renal diseases, thyroid disorders, acute infections, recent stroke, diabetic ketoacidosis, non-ketotic hyperosmolar diabetes and any recent surgery in the last two months. Blood samples were collected after overnight fasting, serum was separated and stored at – 80^o^C until assayed as a single batch.

hsCRP was measured using a turbidimetric assay (Quantex CRP ultra sensitive kits, BIOKIT, S.A., Barcelona, Spain) on auto-analyzer Hitachi 911, (ROCHE diagnostics, Indianapolis, Indiana, USA). The hsCRP kits measured ranges from 0.10 to 20.0 mg/L. 

All our patients underwent left ventriculography and selective coronary angiography. Coronary arteries were imaged by standard views with cranial and caudal positions. Presence of ischaemia was defined on the basis of minimum 50% stenosis in coronary vessels. Gensini scoring system was used to determine the severity of CAD. With the help of this scoring system the percentage of blockage in different coronary vessels at different sites of blockage is calculated and each vessel under consideration is given a score.^16^ Left main coronary artery, left anterior descending artery (LAD), left circumflex (LCx) and right coronary arteries (RCA) were assessed. If there were multiple lesions in the same vessel that was regarded as one-vessel disease. Vessel scoring was also calculated and graded into single, double and triple vessel disease.

***Statistical Analysis: ***We used Statistical Package for Social Sciences (SPSS) version 19, for data analysis. To assess differences in age, blood pressure, TC, LDL, HDL, TG, and BMI Student’s t test was utilized. hsCRP, due to its non parametric distribution, was analyzed by Mann-Whitney U test for two groups and KruskalWalli’s test for more than two groups. A p-value of <0.05 was considered as statistically significant. Spearman’s correlation coefficients were also calculated between Gensini score of CAD severity, vessel scores, hsCRP and lipid profile in all CAD patients.

## RESULTS

**Fig.1:**
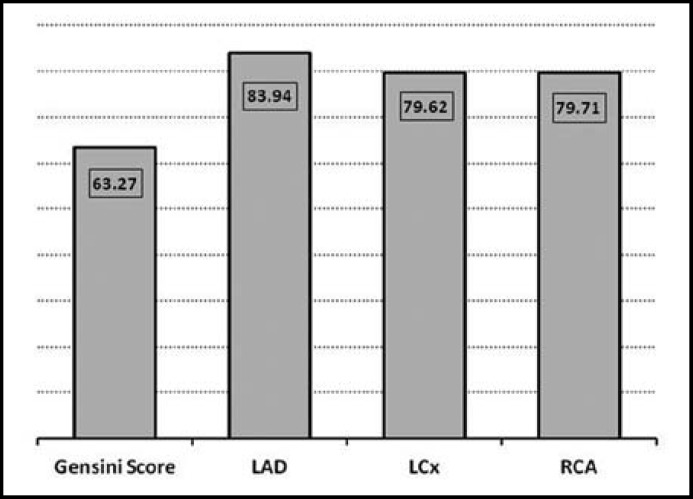
Mean values of Gensini Score and percentage of blockage in LAD, LCx and RCA determined by angiography

**Fig.2:**
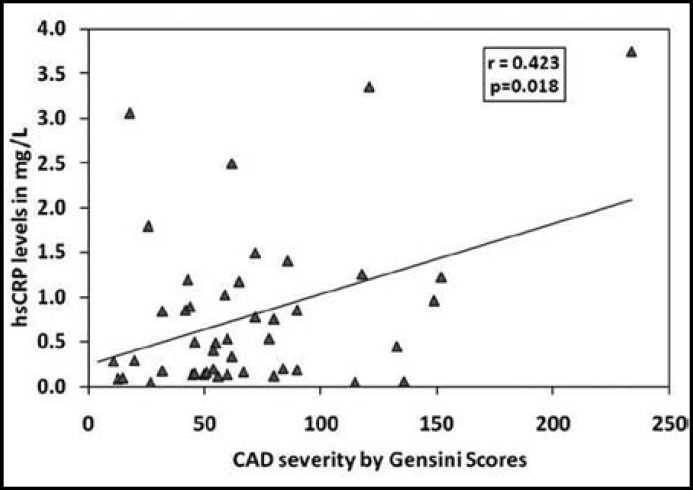
Linear regression analysis between hsCRP and CAD severity determined by Gensini scores in all CAD patients

**Fig. 3:**
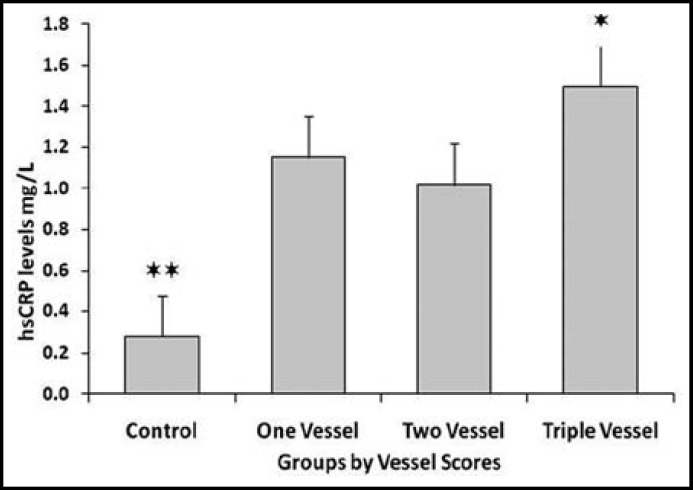
Comparison of mean hsCRP levels in control group and CAD groups according to vessel scores in all CAD patients. *P<0.05 versus one vessel and two vessel CAD groups. ** P<0.01 versus one vessel, two vessel and three vessel CAD groups.

There were non-significant differences in age, weight and BMI among healthy subjects and CAD patients (Table-I). While hsCRP levels were significantly higher in CAD patients compared to healthy individuals. Table-II shows comparison of lipid profile between control and CAD patients. CAD patients had significantly higher TG (p=0.0074) and significantly lower HDL (p=0.0001) levels compared to control subjects. Table-III shows Spearman’s correlations between Gensini score of CAD severity, vessel scores, hsCRP and lipid profile in CAD patients. Although CAD patients presented with higher hsCRP levels but there was no significant correlation of CAD severity with hsCRP or blood lipids. Fig.1. shows mean values of Gensini Score and percentage of blockage in LAD, LCx and RCA determined by angiography. Fig.2. expresses linear regression analysis between hsCRP and CAD severity determined by Gensini scores in all CAD patients and showed a significant positive correlation (r=0.423, p=0.018). We compared hsCRP levels between control group and CAD groups according to vessel scores in all CAD patients. All CAD groups had significantly higher mean values of hsCRP compared to control subjects Fig.3. Triple vessel disease patients had significantly higher hsCRP levels than one vessel and two vessel disease. The difference was non significant between one and two vessel disease groups.

**Table-I:** Clinical characteristics of Control and CAD patients. Values are expressed as Mean±SD

*Variables*	*Control* *n=29*	*All CAD* *n=87*	*P value*
Age years	52.40 ± 8.62	54.83 ± 12.72	0.2883
Height cm	163.81 ± 1.72	162.89 ± 9.19	0.7606
Weight Kgs	74.68 ± 2.64	75.93 ± 15.09	0.9462
BMI	26.69 ± 3.23	28.27 ± 6.21	0.2868
SBP mmHg	125.86 ±15.45	131.97 ± 20.75	0.8256
DBP mmHg	78.7±12.58	76.87 ± 14.65	0.9568
Pulse rate /minute	78.54 ± 7.52	83.73 ± 15.76	0.4841
hsCRP mg/L	0.28 ± 0.32	0.69 ± 0.81	0.0051

Data is expressed as Mean ± SD

Systolic Blood Pressure (SBP), Diastolic Blood Pressure (DBP)

Differences were studied by Mann–Whitney U test for hsCRP and Student's t test for other parameters.

**Table-II:** Comparison of lipid profile between Control and CAD patients

*Analytes mmol/L*	*Control*	*All CAD*	*P value*
TC	4.48 ± 0.60	4.37 ± 1.32	0.6347
TG	1.11 ± 0.49	1.78 ± 1.03	0.0074
LDL	2.76 ± 0.53	2.82 ± 1.11	0.8932
HDL	1.07 ± 0.32	0.75 ± 0.23	0.0001

Data is expressed as Mean ± SD

Total cholesterol (TC), Triglycerides (TG), Low density Lipoprotein (LDL) and

High density lipoprotein(HDL). Differences were studied by Student's t test.

**Table-III:** Spearman’s correlations of hsCRP with CAD severity and lipid profile in CAD patients

	*V Score*	*G Score*	*hsCRP*	*TC*	*TG*	*LDL*	*HDL*
V Score	1.000						`
G Score	.334**	1.000					
hsCRP	.329*	.423**	1.000				
TC	.018	.010	-.089	1.000			
TG	-.160	.053	.032	.422***	1.000		
LDL	.199	.065	-.180	.903***	.192	1.000	
HDL	-.076	.037	-.010	-.469**	.080	-.386**	1.000

*** Correlation is significant at the .001 level (2-tailed).

**Correlation is significant at the .01 level (2-tailed).

*Correlation is significant at the .05 level (2-tailed).

VScore; Vessel Score, G Score; Gensini Score.

## DISCUSSION

The main observations in this study are that hsCRP is a marker of the presence and severity of CAD defined by Gensini scoring or vessel scoring. This can be explained as that hsCRP is an acute-phase reactant protein marker that can demonstrate the subclinical inflammatory states detecting lower serum levels of CRP. There are a lot of advantages in hsCRP measurements related to CAD. One advantage is that it is a stable compound and it can be measured at any time of the day without special relevance to biological clock of the day.^17^ Other markers such as lipids and IL-6, exhibit circadian variations and are related to meals also. Thus, we can perform hsCRP testing in clinical settings without regard for time of day.^18^ Despite all these advantages there is still controversy and limitations of hsCRP levels and other confounding variables as marker of cardiovascular diseases.^19^^-^^22^ Cushman et al have revaluated the prevalence and correlates of increased hsCRP and reported a significant impact of hsCRP measurement on coronary heart disease risk reclassification. They observed that with the inclusion of hsCRP in their testing data, the Reynolds risk score classified the population differently compared to the new Framingham risk scores.^23^ This observation is in agreement to our previous study regarding lipoprotein(a) and its significant correlation with presence, diffuseness and the severity of CAD.^24^

A similar study was performed in Indian population to determine the concentration of hsCRP and its association with coronary atherosclerosis assessed by coronary angiography. In line with our results they reported that the serum concentration of hsCRP was associated with presence of CAD, but regarding severity the correlation was non significant.^25^ It is recently reported that there is state-level geographic variation in inflammatory biomarkers among otherwise healthy women which cannot be completely attributed to traditional clinical risk factors and lifestyle. It is suggested that future research approaches should aim to identify additional factors that may explain geographic variation in biomarkers of inflammation among healthy women.^26^ In a recent study by Hrira et al reported that ApoB and hs-CRP levels were markedly associated with the severity of CAD in Tunisian patients and their findings are similar to our results.^27^ The possible limitations of our study are limited number of subjects and cross sectional design. Prospective studies on large scale are needed to explore the true pathogenic role of hsCRP in assessing cardiovascular risk.

## CONCLUSION

We conclude that patients with angiographically evaluated CAD have significantly higher levels of hsCRP levels compared to healthy individuals and are correlated with the presence & severity of CAD.
